# $^{74}$Ge($n,\gamma$) cross section below 70 keV measured at n_TOF CERN

**DOI:** 10.1140/epja/s10050-022-00878-5

**Published:** 2022-12-09

**Authors:** C. Lederer-Woods, O. Aberle, J. Andrzejewski, L. Audouin, V. Bécares, M. Bacak, J. Balibrea, M. Barbagallo, S. Barros, U. Battino, F. Bečvář, C. Beinrucker, E. Berthoumieux, J. Billowes, D. Bosnar, M. Brugger, M. Caamaño, F. Calviño, M. Calviani, D. Cano-Ott, R. Cardella, A. Casanovas, D. M. Castelluccio, F. Cerutti, Y. H. Chen, E. Chiaveri, N. Colonna, G. Cortés, M. A. Cortés-Giraldo, L. Cosentino, L. A. Damone, M. Diakaki, C. Domingo-Pardo, R. Dressler, E. Dupont, I. Durán, B. Fernández-Domínguez, A. Ferrari, P. Ferreira, P. Finocchiaro, V. Furman, K. Göbel, A. R. García, A. Gawlik-Ramięga, T. Glodariu, I. F. Gonçalves, E. González-Romero, A. Goverdovski, E. Griesmayer, C. Guerrero, F. Gunsing, H. Harada, T. Heftrich, S. Heinitz, J. Heyse, D. G. Jenkins, E. Jericha, F. Käppeler, Y. Kadi, T. Katabuchi, P. Kavrigin, V. Ketlerov, V. Khryachkov, A. Kimura, N. Kivel, M. Kokkoris, M. Krtička, E. Leal-Cidoncha, H. Leeb, J. Lerendegui-Marco, S. Lo Meo, S. J. Lonsdale, R. Losito, D. Macina, J. Marganiec, T. Martínez, C. Massimi, P. Mastinu, M. Mastromarco, F. Matteucci, E. A. Maugeri, E. Mendoza, A. Mengoni, P. M. Milazzo, F. Mingrone, M. Mirea, S. Montesano, A. Musumarra, R. Nolte, A. Oprea, N. Patronis, A. Pavlik, J. Perkowski, I. Porras, J. Praena, J. M. Quesada, K. Rajeev, T. Rauscher, R. Reifarth, A. Riego-Perez, P. C. Rout, C. Rubbia, J. A. Ryan, M. Sabaté-Gilarte, A. Saxena, P. Schillebeeckx, S. Schmidt, D. Schumann, P. Sedyshev, A. G. Smith, A. Stamatopoulos, G. Tagliente, J. L. Tain, A. Tarifeño-Saldivia, L. Tassan-Got, A. Tsinganis, S. Valenta, G. Vannini, V. Variale, P. Vaz, A. Ventura, V. Vlachoudis, R. Vlastou, A. Wallner, S. Warren, M. Weigand, C. Weiss, C. Wolf, P. J. Woods, T. Wright, P. Žugec, n_TOF Collaboration

**Affiliations:** 1 School of Physics and Astronomy, University of Edinburgh, Edinburgh, UK; 2 European Organization for Nuclear Research (CERN), Geneva, Switzerland; 3 University of Lodz, Lodz, Poland; 4 Institut de Physique Nucléaire, CNRS-IN2P3, Univ. Paris-Sud, Université Paris-Saclay, 91406 Orsay Cedex, France; 5 Centro de Investigaciones Energéticas Medioambientales y Tecnológicas (CIEMAT), Madrid, Spain; 6 TU Wien, Atominstitut, Stadionallee 2, 1020 Wien, Austria; 7 Istituto Nazionale di Fisica Nucleare, Sezione di Bari, Italy; 8 Instituto Superior Técnico, Lisbon, Portugal; 9 University of Hull, Hull, UK; 10 Charles University, Prague, Czech Republic; 11 Goethe University Frankfurt, Frankfurt, Germany; 12 CEA Irfu, Université Paris-Saclay, 91191 Gif-sur-Yvette, France; 13 University of Manchester, Manchester, UK; 14 Department of Physics, Faculty of Science, University of Zagreb, Zagreb, Croatia; 15 University of Santiago de Compostela, Santiago, Spain; 16 Universitat Politècnica de Catalunya, Barcelona, Spain; 17 Agenzia nazionale per le nuove tecnologie (ENEA), Bologna, Italy; 18 Istituto Nazionale di Fisica Nucleare, Sezione di Bologna, Italy; 19 Universidad de Sevilla, Seville, Spain; 20 INFN Laboratori Nazionali del Sud, Catania, Italy; 21 Dipartimento Interateneo di Fisica, Università degli Studi di Bari, Bari, Italy; 22 Instituto de Física Corpuscular, CSIC-Universidad de Valencia, Valencia, Spain; 23 Paul Scherrer Institut (PSI), Villigen, Switzerland; 24 Joint Institute for Nuclear Research (JINR), Dubna, Russia; 25 Horia Hulubei National Institute of Physics and Nuclear Engineering, Magurele, Romania; 26 Institute of Physics and Power Engineering (IPPE), Obninsk, Russia; 27 Japan Atomic Energy Agency (JAEA), Tokai-Mura, Japan; 28 European Commission, Joint Research Centre (JRC), Geel, Belgium; 29 University of York, York, UK; 30 Karlsruhe Institute of Technology, Campus North, IKP, 76021 Karlsruhe, Germany; 31 Tokyo Institute of Technology, Tokyo, Japan; 32 National Technical University of Athens, Athens, Greece; 33 Dipartimento di Fisica e Astronomia, Università di Bologna, Bologna, Italy; 34 Istituto Nazionale di Fisica Nucleare, Sezione di Legnaro, Italy; 35 Istituto Nazionale di Fisica Nucleare, Sezione di Trieste, Italy; 36 Dipartimento di Astronomia, Università di Trieste, Trieste, Italy; 37 Dipartimento di Fisica e Astronomia, Università di Catania, Catania, Italy; 38 Physikalisch-Technische Bundesanstalt (PTB), Bundesallee 100, 38116 Braunschweig, Germany; 39 University of Ioannina, Ioannina, Greece; 40 Faculty of Physics, University of Vienna, Vienna, Austria; 41 University of Granada, Granada, Spain; 42 Bhabha Atomic Research Centre (BARC), Mumbai, India; 43 Centre for Astrophysics Research, University of Hertfordshire, Hatfield, UK; 44 Department of Physics, University of Basel, Basel, Switzerland; 45 Australian National University, Canberra, Australia

## Abstract

Neutron capture reaction cross sections on $^{74}$Ge are of importance to determine $^{74}$Ge production during the astrophysical slow neutron capture process. We present new resonance data on $^{74}$Ge($n,\gamma$) reactions below 70 keV neutron energy. We calculate Maxwellian averaged cross sections, combining our data below 70 keV with evaluated cross sections at higher neutron energies. Our stellar cross sections are in agreement with a previous activation measurement performed at Forschungszentrum Karlsruhe by Marganiec et al., once their data has been re-normalised to account for an update in the reference cross section used in that experiment.

## Introduction

Neutron capture cross sections in the keV neutron energy range are an essential input to study nucleosynthesis of the slow neutron capture process (s-process), responsible for forming about half of the elemental abundances between Fe and Bi [1]. The s-process is characterised by a series of neutron captures on Fe seed nuclei, with moderate neutron densities of a few $10^8$ cm$^{-3}$. Since $\beta$-decays proceed typically faster than neutron captures, the reaction path follows along the valley of stability.

Isotopes from mass *A* = 60–90 are produced by the so-called *weak* component of the s-process, occuring in massive stars (more than about 8 solar masses). Neutrons are generated by $^{22}$Ne($\alpha ,n$) reactions during helium core, and later carbon shell burning phases. Germanium is thought to dominantly originate from this stellar site [2], and produced abundances sensitively depend on the value of the Maxwellian averaged capture cross sections, i.e. the neutron capture cross sections averaged over the stellar neutron spectrum. Our collaboration has recently published cross section results on the stable Ge isotopes $^{70,72,73,76}$Ge [3–6]. This article will present results for the remaining stable isotope $^{74}$Ge.

Experimental data of neutron capture reactions on $^{74}$Ge are scarce. In the neutron energy range covered in this experiment (roughly 1 eV to 70 keV), there is only one measurement of resonance properties by Maletski et al. [7], who combined capture and transmission data to obtain resonance parameters. In total, ten resonances have been identified, however for only two resonances it was possible to determine radiative widths. In addition, there is also a transmission measurement of the total cross section of natural germanium by Harvey and Hockaday [8].

There are several measurements of averaged neutron cross sections in the keV region, obtained via the activation technique [9–15]. However, cross sections at neutron energies around 25 keV (where most measurements were performed) show a large scatter from 19 to 54 mb between the existing data (see Fig. 3 of [9]). The scarceness of resonance data, and inconsistencies in activation data, motivated a new measurement of the $^{74}$Ge($n,\gamma$) using the time-of-flight technique at the n_TOF facility.

## Experiment

The measurement was performed at the neutron time-of-flight facility n_TOF at CERN. Neutrons are produced by spallation reactions of a 20 GeV/c proton beam provided by the CERN-PS impinging on a 1.3-ton lead target. The pulsed proton beam has a width of 7 ns r.m.s. and a typical intensity of $7\times 10^{12 }$ protons per pulse, resulting in production of roughly $10^{15}$ neutrons per pulse. The spallation target is surrounded by a water moderator which shapes the initially energetic neutrons to an isolethargic energy spectrum, with neutron energies ranging from a few meV to several GeV. The experiment was performed at a distance of 185 m from the spallation target, at Experimental Area 1, which offers excellent neutron energy resolution ($\varDelta E/E\approx 10^{-3}$ at $E_n=10$ keV) in combination with a high instantaneous neutron flux ($\approx 6\times 10^{5}-1\times 10^{7}$ neutrons per pulse depending on beam collimation). A full description of the facility and neutron beam characteristics can be found in Ref. [16].

The $^{74}$Ge sample consisted of 95.51% isotopically enriched GeO$_2$ with a total mass of 2.575 g. The material was obtained in form of powder and pressed into a cylindrical pellet of 2 cm diameter at PSI Villigen. In addition to the $^{74}$Ge sample, data were also taken with an empty sample holder (to measure the background), a germanium sample of natural isotopic composition (to identify/confirm resonances from isotopic impurities), and a Au sample (for data normalisation) of the same diameter. Backgrounds due to natural radioactivity and cosmic rays were determined in runs without neutron beam.

Radiative capture events were detected with a set of four liquid scintillation(C$_6$D$_6$) detectors , detecting the prompt $\gamma$-rays emitted after a neutron capture. These detectors have been specifically optimised to have an extremely low sensitivity to neutrons scattered off the sample [17, 18], which is essential when studying lower mass nuclei which typically exhibit high neutron scattering-to-capture probabilities.

Data were recorded using 14-bit flash ADCs operated at a sampling rate of 1 GHz. Signal amplitudes and arrival times were determined using an off-line Pulse Shape Algorithm [19].

## Data analysis and results

### Neutron capture yield

Time-of-flight data were converted to neutron energy by calibrating the flight path to resonances of well known energy in $^{197}$Au+n reactions taken from ENDF/B-VIII.0 [20]. The neutron capture yield *Y* can then be obtained as

1 $$\begin{aligned} Y(E_n)=\frac{C(E_n)-B(E_n)}{\varPhi (E_n) \epsilon } \end{aligned}$$

where *C* is the number of counts, *B* is the background, and $\epsilon$ is the efficiency to detect a capture event. The energy dependence of the neutron fluence $\varPhi (E_n)$ was measured in a separate campaign using reference reactions with a well known cross section such as $^{10}$B($n,\alpha$) and $^{235}$U(*n*, *f*) and several different types of detectors [21].

The background *B* was determined in runs with an empty sample holder and in runs without neutron beam to correct backgrounds due to cosmic rays and natural radioactivity.

**Fig. 1:**
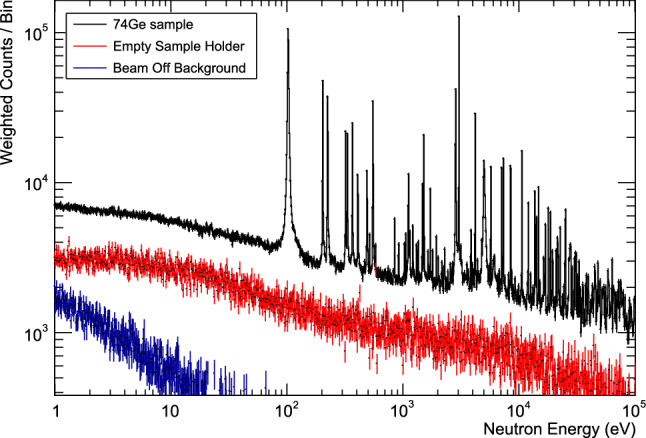
Weighted count spectra from 1 eV to 100 keV neutron energy obtained at n_TOF for the $^{74}$Ge sample, the empty sample holder, and without neutron beam. The resonances below 1 keV come from isotopic impurities, mainly $^{73}$Ge

The detection efficiency typically depends on the $\gamma$-ray energy, which can vary for each neutron capture event. A detection efficiency independent of the de-excitation path of the compound nucleus was achieved by applying the Total Energy Detection principle paired with the Pulse Height Weighting Technique (PHWT) [22, 23], which has been widely used in neutron capture measurements with C$_{6}$D$_{6}$ detectors.

The PHWT is based on applying a pulse height dependent weight to each detected signal, so that, on average, the efficiency to detect a $\gamma$-ray, $\epsilon _\gamma$, is proportional to the $\gamma$-ray energy, $E_\gamma$. If only one $\gamma$-ray of the cascade is detected, the efficiency to detect a capture event $\epsilon$ is then proportional to the excitation energy of the compound nucleus, which is given by the sum of neutron separation energy $S_n$ and centre-of-mass energy $E_{cm}$, hence $\epsilon \propto S_n+E_{cm}$. Polynomial weighting functions were determined by simulations of the detector response to mono-energetic $\gamma$-rays (from 0.1 to 10 MeV) with GEANT4 [24], taking into account the details of sample, detectors and other structural material (detector holders, beam pipes etc.). The data were further corrected for the loss of signals due to the analysis thresholds (200 keV), and due to electron conversion events. These corrections were determined by simulations of realistic neutron capture cascades in $^{75}$Ge and $^{198}$Au using the code dicebox [25].

Figure 1 shows weighted count spectra as a function of neutron energy from 1 eV to 100 keV. The background recorded without neutron beam is only relevant at low neutron energies (corresponding to larger time-of-flight intervals), while the ’empty sample holder’ background due to neutron reactions with the sample holder and other structural material is non-negligible over the entire neutron energy range considered.

**Table 1:** Resonance energies $E_R$ and resonance capture kernels *K* with statistical uncertainties from the fitting procedure

$E_R$(eV)	*K*(meV)	$E_R$(eV)	*K*(meV)
$1518.94 \pm 0.03$	$6.7 \pm 0.2$	$33776.5 \pm 5.2$	$192 \pm 24$
$1729.97 \pm 0.05$	$3.4 \pm 0.1$	$34103.1 \pm 4.1$	$196 \pm 22$
$2858.11 \pm 0.12 ^*$	$151.7 \pm 2.1$	$34768.3 \pm 4.1$	$171 \pm 26$
$3051.40 \pm 0.02 ^*$	$264.9 \pm 3.4$	$34980.9 \pm 0.2$	$51 \pm 39$
$3863.41 \pm 0.10$	$9.1 \pm 0.4$	$35309.5 \pm 7.2$	$224 \pm 34$
$3996.32 \pm 0.50$	$1.9 \pm 0.3$	$35693.2 \pm 5.6$	$335 \pm 41$
$4206.92 \pm 0.04 ^*$	$74.8 \pm 1.4$	$35976.7 \pm 4.6$	$266 \pm 35$
$5022.05 \pm 0.96 ^*$	$258.2 \pm 4.1$	$37559.1 \pm 3.4$	$280 \pm 30$
$5736.94 \pm 0.08$	$52.4 \pm 1.7$	$38164.8 \pm 4.5$	$128 \pm 23$
$7121.27 \pm 0.12$	$75.8 \pm 2.2$	$38271.4 \pm 4.1$	$198 \pm 27$
$7365.95 \pm 0.14$	$129.3 \pm 5.0$	$39699.6 \pm 31.1$	$644 \pm 86$
$8164.51 \pm 0.11$	$3.8 \pm 3.6$	$40112.9 \pm 0.2$	$85 \pm 69$
$8503.52 \pm 0.13$	$272.5 \pm 5.9$	$41607.2 \pm 0.4$	$77 \pm 60$
$10651.12 \pm 0.18$	$289.6 \pm 7.9$	$42612.4 \pm 6.5$	$56 \pm 24$
$12077.96 \pm 0.64 ^*$	$173.7 \pm 7.4$	$43181.4 \pm 28.1^*$	$308 \pm 61$
$12704.85 \pm 0.01$	$17.0 \pm 15.6$	$43638.7 \pm 5.9$	$47 \pm 31$
$13761.38 \pm 0.45$	$232.4 \pm 8.9$	$44481.2 \pm 0.1$	$47 \pm 38$
$13871.82 \pm 0.30$	$81.6 \pm 4.9$	$44745.2 \pm 0.2$	$76 \pm 57$
$14025.08 \pm 5.35$	$12.0 \pm 11.6$	$44992.7 \pm 6.3$	$77 \pm 23$
$14233.75 \pm 0.47$	$147.5 \pm 6.7$	$45197.0 \pm 7.7$	$188 \pm 35$
$14818.55 \pm 0.34$	$252.9 \pm 11.2$	$45405.0 \pm 9.9$	$315 \pm 49$
$16173.12 \pm 2.82$	$10.5 \pm 3.0$	$45653.9 \pm 5.5$	$136 \pm 23$
$16441.78 \pm 0.72$	$68.2 \pm 4.9$	$46044.8 \pm 6.2$	$271 \pm 36$
$16727.72 \pm 2.39$	$11.3 \pm 3.1$	$47233.1 \pm 9.7$	$223 \pm 33$
$16938.33 \pm 0.51$	$178.8 \pm 11.9$	$48011.2 \pm 5.7$	$287 \pm 38$
$17995.66 \pm 0.56$	$242.9 \pm 12.8$	$48214.4 \pm 7.5$	$264 \pm 37$
$18703.53 \pm 1.00$	$151.5 \pm 11.6$	$48616.6 \pm 7.2$	$127 \pm 23$
$18802.10 \pm 2.66$	$56.0 \pm 10.6$	$49183.3 \pm 12.5$	$159 \pm 30$
$18816.82 \pm 1.22$	$82.2 \pm 19.7$	$49727.0 \pm 7.2$	$167 \pm 30$
$19562.03 \pm 3.95 ^*$	$194.6 \pm 18.8$	$50117.1 \pm 9.5$	$116 \pm 29$
$21041.44 \pm 1.12$	$143.3 \pm 14.6$	$50309.1 \pm 7.8$	$397 \pm 47$
$21198.16 \pm 0.71$	$20.9 \pm 18.2$	$52207.1 \pm 6.6$	$281 \pm 35$
$21646.37 \pm 1.06$	$229.6 \pm 13.6$	$52743.0 \pm 5.8$	$126 \pm 22$
$21712.81 \pm 1.49$	$62.4 \pm 5.9$	$53057.2 \pm 9.0$	$218 \pm 36$
$22005.38 \pm 3.84 ^*$	$171.4 \pm 24.9$	$53543.2 \pm 13.8$	$266 \pm 43$
$22819.89 \pm 0.86$	$239.8 \pm 12.6$	$54056.6 \pm 6.5$	$384 \pm 44$
$24532.29 \pm 0.81$	$44.7 \pm 21.2$	$55137.9 \pm 4.9$	$238 \pm 28$
$25063.98 \pm 1.40$	$206.7 \pm 16.0$	$56228.6 \pm 16.0$	$370 \pm 47$
$25191.50 \pm 1.43 ^*$	$225.1 \pm 14.5$	$57237.0 \pm 0.1$	$57 \pm 44$
$25356.25 \pm 3.53$	$101.3 \pm 33.3$	$57961.7 \pm 0.1$	$63 \pm 47$
$25380.40 \pm 1.50$	$348.2 \pm 41.4$	$58242.8 \pm 7.2$	$171 \pm 29$
$27169.58 \pm 1.86$	$125.6 \pm 13.4$	$58859.0 \pm 5.2$	$541 \pm 46$
$27288.62 \pm 1.92$	$163.1 \pm 14.1$	$59375.3 \pm 8.9$	$160 \pm 32$
$27691.80 \pm 2.87$	$284.3 \pm 21.2$	$59584.4 \pm 0.2$	$99 \pm 70$
$27888.79 \pm 2.24$	$134.5 \pm 13.7$	$59760.0 \pm 17.3$	$324 \pm 64$
$28020.09 \pm 1.96$	$223.3 \pm 16.4$	$60167.8 \pm 21.3$	$158 \pm 41$
$28144.00 \pm 3.16$	$42.7 \pm 7.3$	$61354.8 \pm 100.6^*$	$465 \pm 101$
$28962.43 \pm 2.10$	$131.0 \pm 13.5$	$63217.0 \pm 17.3$	$264 \pm 51$
$30187.33 \pm 5.21$	$238.1 \pm 21.4$	$63765.7 \pm 16.0$	$401 \pm 73$
$30879.31 \pm 2.03$	$216.1 \pm 15.7$	$64032.6 \pm 9.5$	$535 \pm 72$
$31227.78 \pm 1.73$	$391.8 \pm 21.5$	$65434.3 \pm 17.6$	$393 \pm 68$
$32263.62 \pm 2.58$	$136.1 \pm 14.1$	$66113.9 \pm 41.0$	$350 \pm 82$
$33172.42 \pm 2.67$	$187.5 \pm 23.6$	$66710.8 \pm 11.6$	$418 \pm 56$
$33225.33 \pm 2.07$	$127.0 \pm 109.9$	$67716.5 \pm 6.9$	$78 \pm 50$
$33352.95 \pm 1.72$	$48.3 \pm 12.2$	$68830.4 \pm 13.5$	$297 \pm 45$

$E_R$ have an additional uncertainty of 0.04% from the uncertainty in the neutron flight path length. *K* has additional uncertainties due to systematic effects of 3% below, and 5.5% above 10 keV (see text for details)

*Resonances observed in a previous experiment [7]

While the pulse height weighting procedure described before allowed to correct for dependencies of the detection efficiency on the de-excitation path of the compound nucleus, this was not sufficient for an accurate determination of the absolute yield, since (i) the distances between detectors and samples are not known with sufficient accuracy, and (ii) the neutron beam was larger in diameter than the capture sample. An accurate normalisation of the yield was achieved using the saturated resonance technique at the 4.9-eV resonance in Au [26]: Measurements were taken with a Au sample of the same diameter as the Ge sample and an areal density large enough, so that essentially all neutrons near the resonance energy are captured, and produce a capture $\gamma$-cascade. This allows normalisation of the data with high accuracy, since there is almost no dependence of the maximum yield on the exact values of the 4.9-eV resonance parameters. The neutron beam size slightly depends on neutron energy. This dependency was corrected using simulations, which have been verified experimentally [16]. These corrections were below 2% in the neutron energy range of interest.

The systematic uncertainty associated with the PHWT is 2% [23]. In addition, we estimate uncertainties due to the threshold corrections, and the normalisation at 4.9-eV to be at most 1%.

### Resonance fitting

The capture yield was analysed using the multi-level R-matrix code SAMMY [27]. Neutron resonances associated with $^{74}$Ge+n were identified by comparing the enriched sample data to data recorded with a metallic Ge sample of natural composition. Resonances were fitted including backgrounds from isotopic impurities, and assuming a constant residual background in the region around the resonance. In addition, Doppler and resolution broadening, as well as multiple scattering and self shielding effects were taken into account in the fitting procedure.

**Fig. 2:**
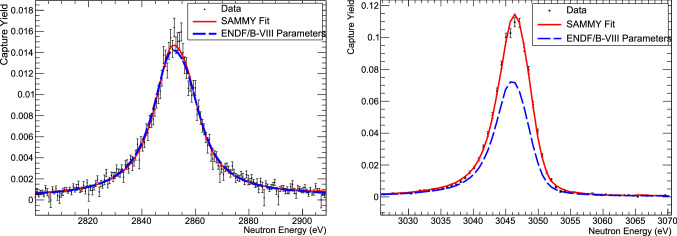
n_TOF data and SAMMY fits for resonances at 2858 and 3051 eV compared to the ENDF/B-VIII evaluation, which is based on a measurement by Maletski et al. [7]

**Fig. 3:**
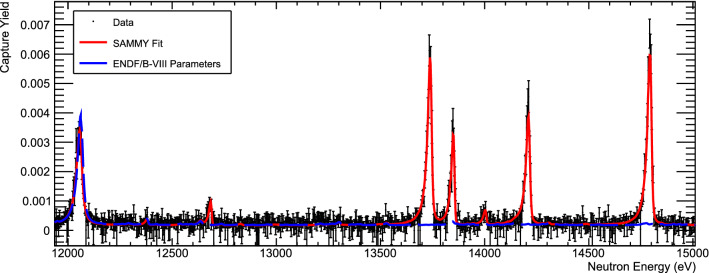
n_TOF data and SAMMY fits for resonances from about 12 to 15 keV, where most resonances are observed for the first time

In general, capture data alone do not allow to determine all individual parameters (resonance spin *J* and parity $\pi$, neutron and radiative widths, $\varGamma _n$ and $\varGamma _\gamma$, respectively). However, for all resonances we can determine their energies $E_R$ and capture kernels *K* defined as

2 $$\begin{aligned} K=g\frac{\varGamma _\gamma \varGamma _n}{\varGamma _\gamma +\varGamma _n} \end{aligned}$$

where *g* is the spin statistical factor

3 $$\begin{aligned} g=\frac{2J+1}{(2s+1)(2I+1)} \end{aligned}$$

with *s* being the neutron spin ($s^\pi =1/2^+$), and *I* the ground state spin of the target nucleus ($I^\pi =0^+$).

The results for capture resonance energies and capture kernels are shown in Table 1 with statistical uncertainties. The uncertainty of resonance energies due to the uncertainty in determining the neutron flight path length is around 0.04%. Uncertainties of the capture kernels due to systematic effects are in total 3% below, and 5.5% above 10 keV, due to the neutron fluence shape (2% below, and 5% above 10 keV), the PHWT (2%), and the normalisation (1%). Fits were performed up to neutron energies of 70 keV, for higher energies the worsening neutron energy resolution and decreasing capture cross section and neutron flux made it too difficult to distinguish resonances from the background.

We can compare capture kernels to the only previous resonance capture measurement for two resonances. Maletski et al. [7] obtained $\varGamma _n=8\pm 2$ eV and $\varGamma _\gamma =0.16\pm 0.04$ eV for the resonance at 2858 eV, with $J^\pi =1/2^+$, which, assuming no correlation between the individual widths results in a $K=157\pm 38$ meV, in good agreement with our result $K=151.7 \pm 5.0$ meV. At 3051 eV, Maletski found $\varGamma _n=1.0\pm 0.6$ eV and $\varGamma _\gamma =0.23\pm 0.04$ eV with $J^\pi =1/2^+$, resulting in $K=187\pm 34$ meV, which is significantly different from our result $K=264.9\pm 8.6$ meV. Figure 2 shows the n_TOF data and SAMMY fits for those two resonances, compared to predictions using ENDF/B-VIII resonance parameters, which are equivalent to Maletski et al., except for a slight adjustment in resonance energy to match Harvey and Hockaday’s [8] total cross section data. In Fig. 3, we show an example of our data and SAMMY fits at higher neutron energies, where our experiment identified a number of previously unknown resonances.

Despite the fact that resonance *J*, $\varGamma _n$ and $\varGamma _\gamma$ can not be determined for all resonances, we can determine at least some of these quantities for several, especially strong resonances. Using the resonance parameters obtained from the fitting, we are able to constrain the average resonance parameters for s-wave resonances, namely, the average radiative width $\langle \varGamma _\gamma ^{\ell =0}\rangle$, the average resonance spacing $D_0$, and neutron strength function $S_0$. For this purpose we assumed that there are no unresolved doublets or even more complex structures.

During determination of the average radiative width we relied on the assumption that the strongest resonances (in terms of $g\varGamma _n/\sqrt{E_n}$) are of *s*-wave character, i.e. $J^\pi =1/2^+$. For all these resonances $k\approx g\varGamma _\gamma$, $g=1$, and $\varGamma _\gamma$ thus should be a reliable quantity. The $\varGamma _\gamma$ from seven strongest resonances below 35 keV (all of them are definitely of *s*-wave origin) yield the average value $\langle \varGamma _\gamma ^{\ell =0}\rangle =211(17)$ meV and the standard deviation of the distribution $\sigma _{\varGamma _\gamma }=44(13)$ meV. Our value of the average radiative width is in excellent agreement with 195(40) meV of Mughabghab [28].

**Table 2:** Maxwellian averaged cross sections of experimental data combined with the evaluated libraries ENDF/B-VIII [20] (same as JENDL-5.0 [30]), JEFF-3.3 [31] and TENDL-2021 [32]

*kT* (keV)	$\langle \sigma \rangle$ (mb)		
	n_TOF+ENDF/B-VIII	n_TOF+JEFF-3.3	n_TOF+TENDL-2021
5	$118.5 \pm 3.6$	$118.5 \pm 3.6$	$118.5 \pm 3.6$
10	$79.7 \pm 3.0$	$79.6 \pm 3.0$	$79.7 \pm 3.0$
15	$63.0 \pm 2.7$	$62.8 \pm 2.7$	$63.4 \pm 2.7$
20	$53.3 \pm 2.4$	$52.8 \pm 2.4$	$53.9 \pm 2.4$
25	$46.8 \pm 2.3$	$46.0 \pm 2.2$	$47.6 \pm 2.3$
30	$42.2 \pm 2.3$	$41.1 \pm 2.2$	$43.1 \pm 2.4$
40	$36.0 \pm 2.6$	$34.6 \pm 2.4$	$37.1 \pm 2.6$
50	$32.0 \pm 2.9$	$30.4 \pm 2.6$	$33.2 \pm 2.8$
60	$29.2 \pm 3.1$	$27.5 \pm 2.8$	$30.5 \pm 3.0$
70	$27.1 \pm 3.2$	$25.4 \pm 2.9$	$28.4 \pm 3.1$
80	$25.5 \pm 3.3$	$23.8 \pm 3.0$	$26.8 \pm 3.2$
90	$24.2 \pm 3.3$	$22.6 \pm 3.0$	$25.6 \pm 3.2$
100	$23.2 \pm 3.4$	$21.6 \pm 3.0$	$24.5 \pm 3.3$

Uncertainties of JEFF-3.3 and ENDF/B-VIII cross sections have been assumed as 20% while the uncertainy for TENDL-2019 is available online [33]

The strongest resonances (in $g\varGamma _n/\sqrt{E_n}$) can be also used for determination of the *s*-wave neutron strength function $S_0$. The exact value strongly depends on the maximum neutron energy used; $S_0$ from maximum energies of 35 and 70 keV yields $\approx 1.0(4) \times 10^{-4}$ and $\approx 2.4(7)\times 10^{-4}$, respectively; the uncertainties are dominated by the Porter-Thomas fluctuations of individual reduced widths. The actual $S_0$ is very likely between these two values; Ref. [28] gives $S_0=2.2(7)\times 10^{-4}$, which was determined from the strongest resonances below 62 keV. To determine $D_0$ we adopted a method similar to that used in the analysis of previous Ge isotopes [3–6]. We compared the observed number of resonances having a kernel higher than a threshold with predictions from simulations based on the statistical model, i.e., assuming Porter-Thomas distribution of reduced neutron widths and Wigner spacing of neighbouring resonances. The $\varGamma _\gamma$ in simulations were assumed to have a common expectation value (given above) for all $J^\pi$ and to originate from a $\chi ^2_\nu$ distribution with $\nu =35$ degrees of freedom; this $\nu$ gives $\sigma _{\varGamma _\gamma }/\langle \varGamma _\gamma \rangle \approx 1/4$, in agreement with the experiment. For the resonance density we further assumed the spin dependence from Ref. [29] and parity independence. Our data give $D_0=3.0(4)$ keV consistently for several different maximum neutron energies and thresholds. The deduced $D_0$ is also virtually independent of the exact values of $S_0$ and $S_1$ used in simulations; for $S_0$ we tested the above-mentioned wide range, for $S_1$ the range $1-3\times 10^{-4}$. Our $D_0$ perfectly agrees with 3.0(8) keV of Ref. [28].

**Table 3:** Maxwellian averaged cross sections obtained from our experimental results in combination with the TENDL-2021 evaluation

*kT* (keV)	$\langle \sigma \rangle$ (mb)		
	n_TOF$+$TENDL-2021	Marganiec et al.	KADoNiS-v1.0
5	$118.5 \pm 3.6$	$106 \pm 12$	106.8
10	$79.7 \pm 3.0$	$70.2 \pm 7.3$	76.8
15	$63.4 \pm 2.7$	$55.0 \pm 5.8$	61.0
20	$53.9 \pm 2.4$	$46.9 \pm 5.0$	51.4
25	$47.6 \pm 2.3$	$41.1 \pm 4.6$	45.0
30	$43.1 \pm 2.4$	$37.6 \pm 3.9$	$40.3 \pm 4.2$
40	$37.1 \pm 2.6$	$32.6 \pm 3.4$	34.1
50	$33.2 \pm 2.8$	$29.0 \pm 3.1$	30.0
60	$30.5 \pm 3.0$	$26.3 \pm 2.8$	27.2
70	$28.4 \pm 3.1$		
80	$26.8 \pm 3.2$	$23.4 \pm 2.8$	23.5
90	$25.6 \pm 3.2$		
100	$24.5 \pm 3.3$	$22.0 \pm 3.0$	21.2

This is compared to results from Marganiec et al. [9], and the latest version of the KADoNiS Database v1.0 [36], which is based on Marganiec et al., but re-normalised to an updated $^{197}$Au($n,\gamma$) cross section, which was used as a reference reaction in that measurement

## Maxwellian averaged cross sections and astrophysical implications

In a stellar environment, neutrons are rapidly thermalised, hence, the neutron capture rate depends on the neutron capture cross section averaged over a Maxwell Boltzmann velocity distribution. This Maxwellian averaged cross section (MACS) at stellar temperature *T* is defined as

4 $$\begin{aligned} \langle \sigma \rangle = \frac{2}{\sqrt{\pi }} \frac{1}{(kT)^{2}}\cdot \int _0^\infty E \sigma (E) \cdot \exp {\left( -\frac{E}{kT}\right) } \text{ d }E \end{aligned}$$

For s-process environments, stellar temperatures can reach up to 1 GK (corresponding to *kT*=86 keV). Hence, to reliably calculate MACSs for all the relevant stellar temperatures, neutron capture cross sections need to be known up to several hundred keV. In this measurement, we determined the cross section up to 70 keV. Therefore, the data need to be supplemented by evaluated neutron capture cross sections for neutron energies above. We have calculated MACSs up to $kT=100$ keV combining our experimental results limited to neutron energies below 70 keV with the major nuclear data libraries ENDF/B-VIII [20], JENDL-5.0 [30] (which is the same as ENDF/B-VIII), JEFF-3.3 [31], and TENDL-2021 [32] above 70 keV. Results are shown in Table 2. The uncertainties for evaluated cross sections have been assumed to be 20% for ENDF/B-VIII / JENDL-5.0 and JEFF-3.3, while the uncertainties for TENDL-2021 can be found online [33]. The contribution of our experimental results to the MACSs is at least 80% up to $kT=30$ keV, and then drops gradually to about 26–30% at $kT=100$ keV. MACSs in combination with the libraries show no large variation, and remain within 14% for all *kT*-values.

To choose which combination to use for our astrophysical impact study, we compare averaged cross sections between 10 and 70 keV obtained from our data to libraries. Overall, the TENDL-2021 evaluation yields the best agreement with our data, therefore, we subsequently use TENDL-2021 for $E_n>70$ keV. Taking into account the uncertainties quoted for the TENDL cross section (e.g. 17.5% around 100 keV [33]), we obtain total MACS uncertainties between 3% at $kT=5$ keV and 14% at $kT=100$ keV. In Table 3, these values are compared to the most recent measurement by Marganiec et al. [9]. In that work, the MACS at $kT=25$ keV was obtained via activation in a quasi-stellar neutron spectrum at Forschungszentrum Karlsruhe, and the MACS values at other temperatures were obtained by extrapolation using the energy dependence predicted by evaluated libraries. The MACS values are 11–16% higher over the entire range of *kT*-values. Table 3 also shows the MACS values recommended by the newest (test) version of the Karlsruhe Astrophysical Database of Nucleosynthesis in Stars KADoNiS-v1.0 [36]. These values are based on results of [9], however they have been re-normalised to an updated $^{197}$Au($n,\gamma$) cross section [37, 38], which was used as a reference reaction in that experiment (see Ref. [39] for details). In addition the extrapolation to other *kT* values was performed differently. The re-normalised MACS at 30 keV is now in agreement with our results within uncertainties.

**Fig. 4:**
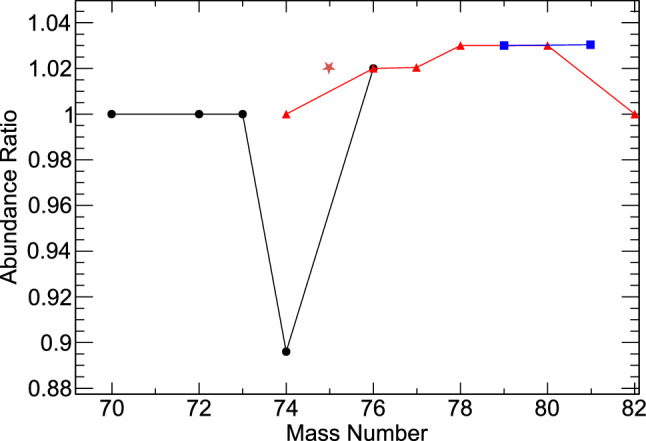
Ratio of abundances for Ge (circles), As (star) , Se (triangles) and Br (squares) isotopes produced after carbon shell burning in a 15 solar mass star with metallicity $Z=$0.006, using $^{74}$Ge MACS of this work, compared to MACS from Marganiec et al. [9]. The lines connect isotopes belonging to the same element

We investigate the impact of our results on nucleosynthesis for the model of a massive star of 15 solar masses and a metallicity of $Z=0.006$ [40]. *s*-process nucleosynthesis was calculated by means of post-processing using the multi-zone code mppnp [41]. The weak s-process in massive stars is activated in two phases of stellar evolution. At the end of helium core burning neutrons are released by $^{22}$Ne($\alpha ,n$) reactions at stellar temperatures around 0.3 GK. The material is then reprocessed during the later carbon shell burning phase at higher stellar temperatures up to 1 GK, again via the $^{22}$Ne($\alpha ,n$) neutron source. For our investigation, we compare final abundances after carbon shell burning using the standard network, which is based on Marganiec et al. [9] data, and our results. Figure 4 shows abundance ratios for the stable isotopes of Ge, As, Se and Br using MACSs of this work, compared to MACSs from Ref. [9] (which are 11–16% lower over all *kT*-values). Our results show a decrease of $^{74}$Ge abundances by about 10%, while the abundances of heavier isotopes along the reaction chain increase by at most 3%.

## Summary

We have measured $^{74}$Ge($n,\gamma$) cross sections at the neutron time-of-flight facility n_TOF at CERN. We obtain in total 110 resonance energies and capture kernels up to 70 keV neutron energy, with systematic uncertainties of 3% below, and 5.5% above 10 keV. Our results are used in combination with the TENDL-2021 evaluation at higher neutron energies to determine Maxwellian averaged cross sections between $kT=5$ and $kT=100$ keV. Our MACSs are in agreement with the most recent measurement by Marganiec et al. [9], once their data have been re-normalised to account for an update in the $^{197}$Au($n,\gamma$) reference cross section [36, 39]. We have studied the impact of our new results on the *weak* s-process occuring in massive stars, using a 15 solar mass model with a metallicity of $Z= 0.006$. Using the new cross sections, the $^{74}$Ge abundances are about 10% reduced after the carbon shell burning phase, while abundances of heavier isotopes along the reaction chain are a few % higher.

## Data Availability

This manuscript has associated data in a data repository [Authors’ comment: The datasets generated during and/or analysed during the current study are not publicly available yet, but are available from the corresponding author on reasonable request.]
